# Educating the Brain to Avoid Dementia: Can Mental Exercise Prevent Alzheimer Disease?

**DOI:** 10.1371/journal.pmed.0020007

**Published:** 2005-01-25

**Authors:** Margaret Gatz

## Abstract

Physicians often recommend to older adults that they should engage in mentally stimulating activity to reduce the risk of dementia. But is this recommendation based on sound evidence?

Physicians now commonly advise older adults to engage in mentally stimulating activity as a way of reducing their risk of dementia. Indeed, the recommendation is often followed by the acknowledgment that evidence of benefit is still lacking, but “it can't hurt.” What could possibly be the problem with older adults spending their time doing crossword puzzles and anagrams, completing figural logic puzzles, or testing their reaction time on a computer? In certain respects, there is no problem. Patients will probably improve at the targeted skills, and may feel good—particularly if the activity is both challenging and successfully completed.

But can it hurt? Possibly. There are two ways that encouraging mental activity programs might do more harm than good. First, they may offer false hope. Second, individuals who do develop dementia might be blamed for their condition. When heavy smokers get lung cancer, they are sometimes seen as having contributed to their own fates. People with Alzheimer disease might similarly be viewed as having brought it on themselves through failure to exercise their brains.

## What Does the Evidence Show?

Three types of evidence are cited to support the idea that mental exercise can improve one's chances of escaping Alzheimer disease.

### Epidemiological studies

Having more years of education has been shown to be related to a lower prevalence of Alzheimer disease in cross-sectional, population-based studies [1] and to a lower incidence of Alzheimer disease in cohorts followed longitudinally [2]. Typically, the risk of Alzheimer disease is two to four times higher in those who have fewer years of education, as compared to those who have more years of education. Other epidemiological studies, albeit with less consistency, have suggested that those who engage in more leisure activities, especially activities that are mentally stimulating, have a lower prevalence and incidence of Alzheimer disease [3,4]. Additionally, longitudinal studies have found that older adults without dementia who participate in more intellectually challenging daily activities show less decline over time on various tests of cognitive performance [5].[Fig pmed-0020007-g001]

**Figure pmed-0020007-g001:**
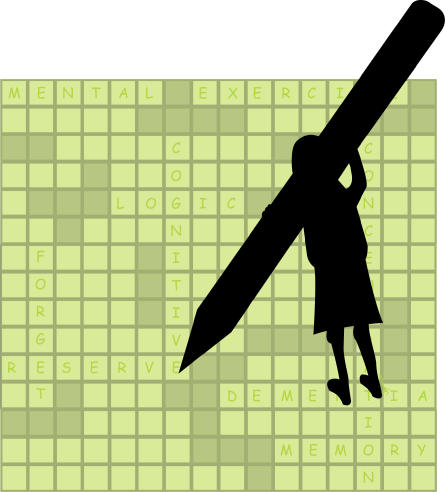
How effective is mental exercise in holding back dementia? (Illustration: Sapna Khandwala, Public Library of Science)

In epidemiological studies, people cannot be randomly assigned to different levels of education, or to different kinds and levels of participation in leisure activities. Consequently, researchers must try to identify confounders and take them into account analytically. However, uncertainties remain. Both education and leisure activities are imperfect measures of mental exercise. For instance, leisure activities represent a combination of influences. Not only is there mental activation, but there may also be broader health effects, including stress reduction and improved vascular health—both of which may contribute to reducing dementia risk [6]. It could also be that a third factor, such as intelligence, leads to greater levels of education (and more engagement in cognitively stimulating activities), and independently, to lower risk of dementia. Research in Scotland, for example, showed that IQ test scores at age 11 were predictive of future dementia risk [7].

Another problem with these epidemiological studies is that reverse causation could be involved—in other words, that incipient dementia could be causing reduced engagement in leisure activities, although some prospective studies have been particularly attentive to controlling for this possibility [8]. Clinical trials are needed to test the hypotheses that emerge from the best epidemiological research. Moreover, because the onset of Alzheimer disease can be hard to pinpoint, and early changes may occur years before the disease is diagnosed, conclusions must be based on large samples, followed over a long period of time.

### Randomized clinical trials

Many studies support the possibility of enhancing memory and other cognitive performance, or of slowing cognitive decline in older adults without dementia [9]. The most effective programs teach mnemonic strategies, provide practice, and give supportive feedback. Mnemonic strategies include the organization of items into meaningful groups, the use of imagery, and the method of loci (visualizing items to be remembered in a sequence of specific, well-learned locations). Comprehensive programs can also include: encouraging memory aids (such as appointment books), teaching relaxation techniques, and providing instruction about memory changes in normal aging. However, improvements are not found in all studies. When improvements are found, they are often modest, may not be maintained over time, and do not generalize beyond the skill being trained. Often, the subjective gains rival the objective ones; for example, participants do tend to report fewer complaints about their memory.

These limitations are evident in one of the largest randomized controlled trials of cognitive training with older adults, a large, multisite study named ACTIVE (Advanced Cognitive Training for Independent and Vital Elderly) [10]. Participants were assigned to receive training in one of three cognitive skills: memory, reasoning, or speed of processing. Tests of cognitive abilities given immediately after training showed large improvements on the particular cognitive skill on which the individual had been trained, but no transfer to the other two cognitive domains. Additionally, for the control group that received no training, simply taking the test battery at pre-test led to improvement on the post-test. The effects of training were maintained over a two-year follow-up. However, the cognitive training program had no significant effect on measures of everyday functioning. Finally, for participants in ACTIVE or in other memory training programs, it remains unknown whether eventual rates of Alzheimer disease will be reduced.

### Neurobiology studies

The third type of evidence suggesting that mental exercise may help to prevent Alzheimer disease comes from neurobiology studies that show greater brain complexity in those with higher levels of mental activity. Many such studies, done with animals, show greater neural complexity after having been exposed to an enriched environment that provides lots of stimulation, for example by including wheels, tunnels, toys, and gnawing sticks [11]. One human study with magnetic resonance spectroscopy showed changes in the hippocampus in elderly memory training participants compared to controls [12]. Another report found changes on positron emission tomography scanning following two weeks of a comprehensive memory program that included memory training, special diet, physical exercise, and stress reduction [13].

## Mental Exercise and Cognitive Reserve

The concept of cognitive reserve is often used to explain why education and mental stimulation are beneficial. The term cognitive reserve is sometimes taken to refer directly to brain size or to synaptic density in the cortex. At other times, cognitive reserve is defined as the ability to compensate for acquired brain pathology. This definition encompasses coping skills as well as recruitment of other brain areas, with cognitive reserve thus accounting for individual differences in severity of cognitive dysfunction when there are pathological neural changes. People with a higher level of education have greater cognitive reserve. In some studies, education or occupation are even used as proxy measures of cognitive reserve, while others are beginning to measure neural substrates that correspond to reserve [14].

Taken together, the evidence is very suggestive that having greater cognitive reserve is related to a reduced risk of Alzheimer disease. But the evidence that mental exercise per se can increase cognitive reserve and stave off dementia is weaker. Epidemiological studies suggest that individual differences in cognitive reserve may actually be lifelong. In addition, people with greater cognitive reserve may choose mentally stimulating leisure activities and jobs, leading to a chicken-and-egg dilemma for the interpretation of the relationship between mentally stimulating activities in adulthood and dementia risk. Cognitive training has demonstrable effects on performance, on views of self, and on brain function—but the results are very specific to the skills that are trained, and it is as yet entirely unknown whether there is any effect on when or whether an individual develops Alzheimer disease. Further, the types of skills taught by practicing mental puzzles may be less helpful in everyday life than more prosaic “tricks,” such as concentrating, or taking notes, or putting objects in the same place each time so that they won't be lost.

## Conclusion

So far, we have little evidence that mental practice will help prevent the development of dementia. We have better evidence that good brain health is multiply determined, that brain development early in life matters, and that genetic influences are of great importance in accounting for individual differences in cognitive reserve and in explaining who develops Alzheimer disease and who does not. At least half of the explanation for individual differences in susceptibility to Alzheimer disease is genetic, although the genes involved have not yet been completely discovered [15]. The balance of the explanation lies in environmental influences and behavioral health practices, alone or in interaction with genetic factors.

For older adults, health practices that could influence the brain include sound nutrition, sufficient sleep, stress management, treatment of mood or anxiety disorders, good vascular health, physical exercise, and avoidance of head trauma. But there is no convincing evidence that memory practice and other cognitively stimulating activities are sufficient to prevent Alzheimer disease; it is not just a case of “use it or lose it.”
